# Quality Characteristics of Poultry Products Containing Plant Components with Enhanced Health Benefits

**DOI:** 10.3390/foods14244307

**Published:** 2025-12-14

**Authors:** Anna Augustyńska-Prejsnar, Małgorzata Ormian, Jadwiga Topczewska, Zofia Sokołowicz, Renata Tobiasz-Salach

**Affiliations:** 1 Department of Animal Production and Poultry Products Evaluation, Faculty of Technology and Life Sciences, Institute of Food Technology and Nutrition, University of Rzeszów, 35-959 Rzeszów, Poland; aaugustynska@ur.edu.pl (A.A.-P.); mormian@ur.edu.pl (M.O.); zsokolowicz@ur.edu.pl (Z.S.); 2 Department of Crop Production, Faculty of Technology and Life Sciences, University of Rzeszów, Zelwerowicza 4 St., 35-601 Rzeszów, Poland; rtobiasz@ur.edu.pl

**Keywords:** quality, nutritional value, poultry product, plant components, sensory characteristics

## Abstract

An innovative approach to improving the quality of meat products is to change their recipe composition. The aim of the study was to improve the quality of poultry products with different proportions of plant components. The test groups consisted of paste products: P_1_—with 50% slaughter turkey meat and 40% plant additives; P_2_—with 30%, respectively. The control group consisted of classic poultry pâté in paste form. The assessment of paste quality considered the physical characteristics (pH, color), nutritional value (basic chemical composition, fatty acid profile, fiber content, vitamin E, cholesterol, minerals), microbiological quality (total number of aerobic bacteria, *Pseudomonas*) and sensory quality of the samples. It was found that poultry products containing plant components had increased nutritional value, including reduced fat and cholesterol content, while maintaining a favorable fatty acid profile, increased fiber, vitamin E and mineral content (Mg, Mn, K, Na, Ca, Fe) as well as microbiological safety and acceptable sensory characteristics compared to the control group. Within the research groups, the product from group P2, with a 60% share of plant components, received a higher recommendation with regard to health-promoting properties (higher fiber, Mn, Mg, Na, Ca, Zn content, optimal ratio of omega 6 to omega 3 fatty acids) and sensory characteristics (tastiness, spreadability).

## 1. Introduction

Promoting health through conscious nutrition is becoming a new dietary trend [1,2]. In response to consumer needs, the meat industry is taking steps to change its range of processed products [3,4,5,6]. In the era of the development of healthy eating trends, the interest in red meat decreases, and the consumption of poultry meat, including turkey meat, is constantly growing [7]. Turkey farming in the European Union (EU) is a well-established industry that plays a significant role in the region’s agricultural sector. Turkey meat is, among other things, a source of complete and easily digestible protein, B vitamins and a number of microelements essential for metabolic processes in the human body. Other compounds contained in meat that are important for the proper functioning of the human body include carnosine, glutathione and choline [8,9,10]. However, high consumption of meat and meat products is associated with health, social and environmental concerns, including animal welfare issues, which has resulted in reduced consumption [2]. In line with current trends, the meat industry is seeking technological solutions that will lead to the production of high-quality meat products. A new direction in shaping the quality of meat products is to change their recipe composition by adding appropriately selected plant components containing bioactive ingredients, antioxidants (e.g., phenolic compounds, flavonoids, tocopherols) and fiber. The use of plant ingredients can significantly improve the nutritional value of meat products, reduce their calorie and cholesterol content, and extend their shelf life [2,5,11,12,13]. In addition to cereals, pseudo-cereals, oilseeds, mushrooms, and protein extracts, legumes and vegetables are suggested in the literature as plant-based ingredients that can substitute meat in meat-based products [2,14,15,16,17,18,19]. The effect of adding legume flours to meat-based products on their quality has been the subject of research for several decades, and there is currently interest in research on the alternative use of legumes and ‘superfoods’ vegetables in the meat processing. Legumes, including chickpeas, have high nutritional value. Chickpeas are a very good source of complete protein, containing essential amino acids and dietary fiber, with a content of 18–20 g per 100 g of dry chickpeas. In addition, they are rich in unsaturated fatty acids, such as linoleic and oleic acids, and are also a source of vitamins A, C, E and B, as well as minerals. Due to the functionality of its components, such as proteins, starch, polyphenols and carotenoids, chickpeas are used as a health-promoting ingredient in many food products [20,21]. Chickpeas are a valued component of meat-plant products because their protein has key characteristics for creating a meat-like structure. These include gelation, emulsification, and water binding properties, which enable hybrid products to achieve the desired juiciness, elasticity, and thermal stability [22,23]. This raw material also has a mild, neutral flavor profile, which makes it easy to combine with meat raw materials and does not require intensive flavor masking, which distinguishes chickpeas from, for example, peas or soybeans. In terms of nutrition, chickpeas provide high-quality protein, fiber, and carbohydrates, and are less allergenic than popular alternatives, which broadens the group of potential consumers [13,20,21,24]. In addition, it is an economically accessible raw material, relatively stable in price, and in line with sustainable development trends. Chickpea pods have a lower environmental footprint and are growing in consumption in Europe. All these features make chickpeas an effective, functional, marketable, and affordable ingredient in the design of modern meat-plant products [14,15,25].

Red peppers are highly valued vegetables. The unique properties of peppers result from the presence of complex alkaloids in the fruit, including capsaicinoids, which give them their specific characteristics: a pungent taste and biological activity. Red peppers contain carotenoids (lycopene, provitamin A), vitamins C and E, flavonoids, phenolic compounds and minerals, mainly calcium and iron. In addition to its culinary qualities, this plant also has valuable medicinal properties. The rich complex of active compounds in peppers determines their antioxidant effect, including other biological activities [26,27,28,29].

Dried tomatoes have greater nutritional value than fresh fruit. They are a rich source of potassium, magnesium and zinc, including vitamins C and A, as well as B vitamins (B_1_, B_2_, B_6_). The most important compound contained in tomatoes, that determines their therapeutic properties, is lycopene [30,31,32,33,34]. Common garlic contains numerous sulphur compounds, which are largely responsible for its biochemical properties. Among the most important sulphur-containing compounds are: alliin (S-allyl cysteine sulfoxide), allicin (diallyl thiosulfinate), allyl methanethiosulfinate, diallyl disulphide, diallyl trisulphide, allyl methyl trisulphide, S-allyl mercaptocysteine, ajoene, S-allyl cysteine. The medicinal and therapeutic uses of garlic are determined by the microelements and vitamins it contains, as well as macronutrients, including 17.5% oligosaccharides with prebiotic properties (e.g., sinistrin) [35,36,37,38]. Black fermented garlic is obtained by fermenting (aging) whole garlic bulbs at a temperature of 60–90 °C with a humidity of 70–95% for a period of several to even 90 days, depending on the intended use of the product. Thermal processing, including enzymatic and non-enzymatic processes cause changes in the content of organic sulfur compounds, an increase in the content of polyphenols, melanoidins, antioxidant activity, polysaccharides, simple sugars, and total acids, as well as the breakdown of fructooligosaccharides and the cellulose structure of the raw material, thereby increasing the availability of bioactive compounds. In addition, it contains, among others: carbohydrates, dietary fiber, protein, fat, minerals including potassium, phosphorus, magnesium, iron, iodine, and selenium, vitamin C, vitamins A and D, B vitamins (B_1_, B_2_, B_3_), and polyphenols. Black garlic has antioxidant properties [39,40,41,42]. Flaxseed oil is distinguished by its highest content of omega-3 fatty acids and very high content of unsaturated fatty acids in relation to other components. Flaxseed oil contains 58% α-linolenic acid, 15% linoleic acid, 18% oleic acid, 6% palmitic acid, 3% stearic acid, and vitamins E and A. Flaxseed oil also contains plant sterols, including β-sitosterol, campesterol, avenasterol, stigmasterol, and carotenoids, which have anti-inflammatory properties [43,44,45,46]. Hemp oil is obtained from the seeds and flowers of Cannabis sativa L. Its health-promoting properties result from its unique composition. Hemp is a rich source of lipids, including linoleic acid (LA; n-6) and alpha-linolenic acid (ALA; n-3). Hemp seed oil contains tocopherol isomers: beta-tocopherol, gamma-tocopherol, alpha-tocopherol, and delta-tocopherol, with gamma-tocopherol being present in the highest amount. Tocopherols are natural antioxidants that can reduce the risk of disorders associated with oxidative degeneration. In addition, it contains polyphenols, which are responsible for the taste of hemp seed oil and its antioxidant activity. Among the phenolic compounds, flavonoids are the most abundant. The quality of hemp oil is determined by its mono- and sesquiterpene content. Alongside cannabinoids, the terpenes contained in the oil determine its anti-inflammatory and antioxidant properties [47,48,49].

Thanks to the development of new, enriched meat products, the range of foods with functional properties is expanding. The balance between the technological challenges and health benefits of an enriched product should be balanced by the organoleptic properties of the finished product [12,13,50,51].

The aim of the study was to evaluate the quality of a poultry product in the form of a paste with varying proportions of plant components with health-promoting properties.

## 2. Materials and Methods

### 2.1. Ingredients and Recipe Composition

The meat-based raw material consisted of breast muscles from slaughtered turkeys, without skin and bones, and pork jowl, which were purchased in a single transaction at a butcher’s shop in Rzeszów, Poland. The poultry meat came from the same producer and had a valid use-by date. The meat-based raw material in its original airtight packaging was transported in portable refrigerators (4 ± 1 °C) to the laboratory of the Institute of Food Technology and Nutrition at the University of Rzeszów. The raw material was then divided into two equal portions, sealed in vacuum packaging (with a raw material weight of 0.5 kg) and stored frozen in a −20 ± 2 °C freezer (Liebherr, Ulm, Germany) for a period of 7 days (first series of research) and two weeks (second series of research).

For the purposes of the study, an innovative recipe for selecting raw materials was developed by increasing the proportion of non-meat raw materials in poultry-based products, i.e., using plant components that are a rich source of nutrients and bioactive compounds with health-promoting properties. The plant ingredients were: chickpeas, red peppers, linseed oil, hemp oil, fermented black garlic, dried tomatoes, common garlic and common onions. The research groups were differentiated by the proportion of roasted chickpeas, while the other plant components were kept in constant proportions (Table 1). Gluten-free chickpeas from Bio Planet, certified as an organic product PL-EKO-07; BIG Nature cold-pressed linseed oil, obtained from organically grown seeds; Bio Planet cold-pressed organically grown hemp seed oil and Juleko Bio fermented black garlic; Nat Vita dried tomatoes were purchased from an organic food store. Vegetables: peppers, onions and garlic bulbs from a local producer, other ingredients such as natural non-iodized salt ‘Kłodawska’, black pepper from ‘Dary natury’ from a health food store.

### 2.2. Preparation of Poultry Products Containing Plant Components

Two experimental series were conducted at 7-day intervals, in which three product variants were produced according to the same production scheme. The recipe composition of the pastes is shown in Table 1. The control group (P_M_) consisted of a turkey meat paste based on the recipe for classic poultry pâté (spreadable). In the experimental groups, the recipes were reformulated by replacing the meat raw material with plant ingredients (chickpeas, linseed oil, hemp oil, red pepper, garlic, dried tomatoes, fermented black garlic). In the meat and vegetable paste from group P1, the turkey meat content was 50%, while in the meat and vegetable paste from group P_2_, the turkey meat content was 30%. In each series, before proceeding with the production of meat and vegetable pastes, the meat raw material was thawed at 4 °C in an FKv36/10 refrigerator (Liebherr, Ulm, Germany) until the internal temperature of the raw material reached 4 ± 1 °C. The turkey breast and pork jowl meat were cut into 300 ± 20 g portions. They were steamed in water in separate containers at 90 ± 5 °C until they reached a temperature of 76 ± 4 °C (temperature meter) (Testo104, Lenzkirch, Germany) inside the container. The meat was cooled to 20 ± 2 °C, then minced twice in a meat grinder (Zelmer, Rzeszów, Poland) with a 4 mm mesh. In order to eliminate antinutrients, improve digestibility, and enhance the bioactive and physicochemical profile of chickpeas, they were pretreated. The treatment consisted of rinsing them in tap water and then soaking them in cold water for 12 h. After rinsing and draining again, they were heat-treated by steaming in water using a BSK792320M convection oven (Electrolux, Warsaw, Poland) with a steam function (chamber temperature 100 °C, time 60 min). The steamed grains were left to cool slowly at room temperature (20 ± 2 °C). The steamed chickpea grains were then ground in a VCB-32 cutter (country of origin: Poland) to a particle size of 0.02 ≤ Φ ≤ 0.50 mm. This treatment was aimed at increasing the bioavailability of nutrients, increasing the specific surface area of the particles, and obtaining a more homogeneous mass structure. Sweet red peppers, onions in their skins, and garlic bulbs were heat-treated (baked in a convection oven at 180 °C) for 20 min and cooled to 20 ± 2 °C. The steamed chickpeas and vegetables (peppers, onions, garlic) were ground in a VCB-32 cutter (Hallde, Kista, Sweden) to a particle size of 0.02 ≤ Φ ≤ 0.50 mm. In each group, all ingredients were placed in a mixer with a stainless steel stirrer (Titanium, Havand, UK) and mixed for 5 min until evenly distributed. The procedure for preparing the ingredients was the same in each research group. The prepared stuffing was placed in sterilized glass jars (0.35 L capacity) and preserved by pasteurization (Sencor SPP 1800W pasteurizer, Ricany, Czech Republic) for 40 min and cooled to room temperature. The jars with the paste were stored under refrigerated conditions at 4 ± 2 °C (Liebherr LKPv 6527 MediLine laboratory refrigerator, Ulm, Germany). After 12 h of storage, the meat and vegetable pastes were tested for physical, chemical, and microbiological characteristics and sensory evaluation. The microbiological quality assessment test was performed after 48 h of refrigerated storage.

### 2.3. Assessment of Physical Properties

The pH value of poultry products was measured using a HI 99163 digital pH meter (Hanna Instruments, Woonsocket, RI, USA) equipped with an FC232 composite electrode (Hanna Instruments, Woonsocket, RI, USA). Before the measurements, the pH meter was calibrated using the two-point method with pH 4.01 and pH 7.01 reference buffers (Hanna Instrument Company, Salaj, Romania). The measurement was performed by inserting 2/3 of the electrode height into the product and reading the results from the device. The measurements were performed in three replicates for each sample, and the procedures were the same for all samples. The color of poultry products was measured using a Chroma Meter colorimeter (Konica Minolta, Osaka, Japan) with a CR-400 head (ø = 11 mm), CIE L*a*b system, D56 light source, and 2° standard Observer. The color was measured by placing the device’s head on the surface of the meat sample being tested. The results were read from the device display. This method allowed for the evaluation of color parameters that describe the brightness of the tested samples (L* parameter) and their chromatic colors (a* parameter—red and b* parameter—yellow). Three measurements were taken for each sample, and the test procedure was always the same. The test was carried out in a lit room at a room temperature of 20 ± 2 °C.

#### Assessment of Chemical Traits

The protein content in poultry products was determined using the Kjeldahl method for nitrogen content determination. Appropriately prepared samples were weighed with an accuracy of 0.0001 g in mineralization tubes. Two parallel determinations were performed for each sample. The sample was mineralized in a mineralization block with controlled temperature regulation. The steam distillation process was carried out in an automatic apparatus—BÜCHI Labortechnik AG (Flawil, Switzerland). Distillation involves adding excess sodium hydroxide to the cooled mineralized sample to release ammonia. The released ammonia was distilled with steam into excess boric acid, and the resulting solution was titrated with hydrochloric acid using an electronic burette until the color changed. The amount of nitrogen in the sample was calculated based on the volume of HCl used for titration. The calculated amount of nitrogen determined in the test sample was converted to protein using a nitrogen-to-protein conversion factor of 6.25.

The free fat content in raw materials and poultry products was determined using the extraction weighing method (Soxhlet-Soxtherm extraction apparatus from Gerhardt, Königswinter, Germany), with an electric dryer capable of maintaining a temperature in the range of 103 ± 2 °C, petroleum ether with a boiling point of 40 to 60 °C.

The composition (percentage of fatty acids in total fatty acids) in poultry products was determined using the DGF C-VI 11a: 2016 mod +DGF C VI 10a: 2016 mod method (Agilent Technologies 7890A GC system with FID Detector and a CP-Sil 88 Säule from Agilent, Santa Clara, CA, USA). The principle of the method was based on the separation of fatty acids (identification of fatty acids after retention time) using gas chromatography with flame ionization detection. Gas chromatography of fatty acid methyl esters was performed using capillary gas chromatography with flame ionization detection. The transesterified sample solution was distributed through a gas chromatograph by injection with stream splitting on a CP-Sil column and analyzed by a flame ionization detector (Agilent Technologies, Inc., Santa Clara, CA, USA).

The total ash content was determined by the gravimetric method (Nobetherm P330 muffle furnace, Lilienthal, Germany).

The total dietary fiber in the poultry product was determined using an enzymatic-gravimetric method (Kjeldatherm mineralization block from Gerhardt, Königswinter, Germany, with controlled temperature regulation, Vapodest Carousel automatic distiller from Gerhardt, Germany, vacuum filtration kit from Foss Analytical A/S, Hillerød, Denmark). Samples were dried overnight and defatted with petroleum ether, then ground to particles smaller than 0.3 mm. MES/TRIS buffer, c = 0.05 mol/L, was added and adjusted to pH 8.3. The samples were incubated with successive additions of α-amylase, protease, and amyloglucosidase solutions. After the enzymatic decomposition stage, the sample was poured with heated ethyl alcohol (78%) to precipitate the sediment and filtered under vacuum through a glass filter crucible. The crucible with sediment was dried for 12 h at (103 ± 2) °C and then weighed. In one crucible, the ash content was determined by roasting in an oven at 550 ± 25 °C, while in the other, nitrogen was determined in the same way as for protein determination. The dietary fiber content in the test sample was calculated from the values obtained.

Vitamin E (DL-alpha-tocopherol acetate and tocopherol) in the products was determined using REG (EC) 152/2009, IV, B: 2009-02 [52].

The microbiological quality assessment (the colony-forming units per gram, *Pseudomonas* Spp.) was carried out after 48 h of refrigerated storage of the products (5 °C). Using a sterile scalpel, 10 g of poultry product was collected from each test group and transferred to sterile Petri dishes. The samples were homogenized in 90 mL of diluent (0.9% NaCl + 0.1% peptone) in a BagMixer^®^ laboratory homogenizer (stomacher) and serial dilutions of 10^−2^–10^−5^ were performed. The cultures were made on PCA medium (Biocar) and incubated at 30 °C for 72 ± 3 h. The total number of bacteria was determined in the samples (log cfu·g^−1^) as colony-forming units per gram. For *Pseudomonas* Spp. identification, the procedure was similar in the first stage. Using a sterile scalpel, 10 g of poultry product was taken from each test group and transferred to sterile Petri dishes. The samples were homogenized in 90 mL of diluent (0.9% NaCl + 0.1% peptone) in a BagMixer^®^ laboratory homogenizer (stomacher) and serial dilutions of 10^−1^–10^−5^ were performed. The cultures were made on CFC agar base and incubated at 25 °C for 44 ± 4 h. Five selected typical colonies were confirmed with an oxidase test. The number of *Pseudomonas* spp. bacteria (log cfu·g^−1^) was determined in the samples.

### 2.4. Sensory Evaluation

The sensory evaluation of the meat plant pastes was carried out in accordance with the international standards for selection, training, and sensory analysis. A trained sensory panel consisting of 10 people was previously recruited from among scientists working at the Institute of Food Technology and Nutrition at the University of Rzeszów. The panel had previous experience in sensory evaluation of poultry meat and other meat products. Before the main study, a preliminary test was performed during which the panelists were familiarized with the identification of sensory attributes necessary to describe odor, flavor, color desirability, connection, juiciness/moisture, consistency/structure, spreadability, and general acceptability. Sensory evaluation was performed using a five-point hedonic scale. For the purposes of the study, three samples of each product were prepared for each evaluator and coded. The samples were presented to the evaluators in white containers with transparent lids, which were placed on white trays. Right before the evaluation, the containers with the samples were opened, and the panelists received mineral water to rinse their mouths. The procedure was the same for each sample. The study was conducted in a specially prepared room free of foreign odors and any distracting factors, under controlled lighting, temperature, and humidity conditions PN-EN ISO 8589:2010 [53].

### 2.5. Statistical Analysis 

The results of the study are presented using arithmetic means ($\bar{x}$) and standard deviation (SD). The distribution of variables was examined using the Shapiro–Wilk test, and the Levene test was used to determine the homogeneity of their variance. Statistical analysis of the results was performed using one-way analysis of variance (ANOVA). Tukey’s test was used as a post hoc test to determine the differences between the test series. A probability of *p* ≤ 0.05 was considered statistically significant. The calculations were performed using the Statistica software package 13.3 [54].

## 3. Results and Discussion

Our research has shown that the pH value of poultry products containing plant components was higher than that of the control group (*p* ≤ 0.05) (Table 2). Within the research groups, no statistically significant differences were found in the degree of acidification of poultry products containing plant components. The results obtained may have been due to the pH of the components used, with steamed chickpeas accounting for the largest share. The results are consistent with those obtained by Motamedi et al. [25] and Kasaiydan et al. [15] using various forms of chickpeas in minced meat products. In the study by Abbullach and SakranAbass [55], it was noted that as the proportion of cooked chickpeas increased, so did the pH of veal burgers. The increase in pH of ready-made meat and plant products containing plant components was due to the high proportion of cooked chickpeas, which are the main alkalizing factor in the formulation. Chickpeas are characterized by a high content of alkaline amino acids, in particular arginine and lysine [51]. The presence of these amino acids contributes to an increase in the pH of the product, as their alkaline side chains can bind protons and reduce the concentration of hydrogen ions in the protein-plant matrix. In addition, the process of cooking chickpeas significantly modifies the chemical composition of the raw material. During heat treatment, some of the soluble organic acids, including phenolic acids, malic acid, and small amounts of citric acid, are washed out into the water, which leads to a decrease in the acidity of the raw material. As a result, cooked chickpeas have a higher pH than raw chickpeas, and their addition to meat-plant product formulations further shifts the pH of the entire system towards less acidic values. This effect is enhanced by the loss of buffering acids as a result of leaching, which reduces the buffering capacity of the product and increases its susceptibility to pH increase [15].

Color modifications with respect to the original meat product directly affect the quality and acceptability of the food, as color is probably the most influential characteristic of meat products [54]. In addition to meat raw materials, plant additives and the proportions of ingredients used in the recipe can play important roles in modifying the color of meat-plant products [14]. The results of the color assessment of meat-plant pastes showed that products with plant additives, in all tested groups, were characterized by a lower (*p* ≤ 0.05) L* brightness parameter (darker color) and a higher degree of red and yellow color saturation. The color changes in meat-plant products compared to poultry meat are mainly due to the presence of plant pigments such as carotenoids (β-carotene, capsanthin), lycopene, and flavonoids, which come from chickpeas, peppers, and tomatoes, among other sources. Color stability during storage and heat treatment is determined both by the chemical structure of these pigments and their interactions with proteins, carbohydrates, and fats in the product matrix [32,56,57,58,59,60,61]. High temperature, oxygen, and oxidation processes can lead to the degradation of carotenoids and lycopene, resulting in changes in L, a, and b* values and an observable darkening or shift in color towards yellow-red. In addition, Maillard reactions and the formation of melanoidins in the presence of sugars and amino acids may have further influenced the hue and intensity of the final color of the poultry product containing plant components (P_1_ and P_2_) that differed significantly across the color spectrum. The products from the test groups were darker, redder, and yellower due to the presence of carotenoids and lycopene from peppers and tomatoes, as well as melanoidin pigments formed during processing. In general, any color changes observed in meat products containing paprika and tomatoes are mainly due to the addition of these ingredients, rather than the color of the meat [62,63]. Paprika has a high carotenoid content. The ketocarotenoids capsanthin and capsorubin (red xanthophylls) are the main red compounds primarily responsible for increased a* in the pastes containing paprika [26,27]. Within the research groups, the product from group P_2_, containing 40% steamed chickpeas and the same proportion of other plant components, was characterized by greater yellowness and a darker color.

The change in color was probably due to the high proportion of chickpeas. However, the specific effect of chickpea addition on the paste’s color is difficult to predict since several complex factors are involved. Such factors include the pigment levels of chickpeas and other ingredients, pigment changes during product processing, as well as the paste microstructure’s impact on light scattering [15,64]. Kasaiyan et al. [65] have, in their studies, shown increases in the proportion of yellow color in pork cutlets, while Kasaiyan et al. [15] indicated similar results in lamb sausages with cooked chickpea additions.

The nutritional value of any food is determined by its protein, fat, vitamin, and mineral content. In the case of meat products, however, it is possible to improve their health-promoting properties by reformulating recipes. Legumes, as a source of high protein content, are also perceived as clean-label ingredients that can benefit meat products [14]. Protein is one of the basic nutrients provided by food portion. It is essential for the proper functioning of the body. The current study has demonstrated that the protein content in reformulated products containing plant components was statistically similar (*p* ≤ 0.05) to that in the control product (Table 2). In studies by Kasaiyan et al. [64], no significant differences were found in the protein, lipid, and moisture content of reformulated pork cutlets with 25% cooked chickpea paste. In studies by Motamedi et al. [25] as well as Asmare and Admassu [65] reported an increase in the protein content of meat products with the addition of chickpea and lentil flour.

In our own research, the modification of the recipe resulted in a significant reduction in fat content. In product P_1_, with 40% plant components, the fat content was reduced by over 32%, while in product P_2_, with 60% plant components, by 37% (Table 3). A major advantage of poultry products with plant components, research groups P_1_ and P_2_, was their favorable fatty acid profile. The reformulated products were characterized both by lower contents of saturated fatty acids (SFA) and a higher proportion of polyunsaturated fatty acids (PUFA). The appropriate selection of plant components in the reformulated products allowed for the preservation of a favorable omega-6 to omega-3 ratio compared to the additive-free meat equivalent, thus enhancing their nutritional value. Consuming too much omega-6 inhibits the action of omega-3. Our research has shown that, of the test groups, product P_2_ with a higher proportion of plant components, had a more favorable ratio of omega-6 to omega-3 fatty acids (1:1) (Table 4). This fatty acid profile is optimal from the nutritional point of view and may help prevent lifestyle diseases. The omega-6 to omega-3 ratio was unfavorable (8:1) in the control product. Studies confirming the beneficial effect of plant components, including chickpeas, tomatoes, peppers, linseed oil, and hemp oil, on fat content and fatty acid levels, were also demonstrated in studies by Skwarek and Karwowska [66], Karslıoğlu et al. [67], Castro et al. [60], Al-Madhagy et al. [43], Dąbrowski et al. [44], Mueed et al. [45], Nowak and Jeziorek [46].

Dietary fiber is a dietary component that has beneficial effects on human health. The current noticeable trend towards increasing fiber content in meat products, is related both to the need to improve products’ nutritional value and to consumers’ growing interest in foods containing fiber [13,51,60,65,66]. Our own research has shown that the addition of plant components significantly increased the fiber content from 1.01% in the control group (classic minced meat pâté containing wheat bread) to 3.08% in group P_1_ and 4.44% in group P_2_ (Table 3). Confirmed increases in fiber content have been demonstrated in many studies on the enrichment of meat products with plant components [65], including those containing chickpeas [25,68]. However, it is important that meat products enriched with plant ingredients maintain an adequate level of other nutrients, besides increasing their dietary fiber content [60,69,70].

Vitamins are organic compounds that do not provide energy or serve as structural components of tissues, but are essential for proper growth and development of the body. Among the vitamins found in meat products, vitamin E, which is a natural antioxidant, deserves special attention. It prevents the oxidation of polyunsaturated fatty acids, the consumption of which should be correlated with adequate intake of α-tocopherol. Our research has shown that the content of vitamin E as alpha-tocopherol in products containing plant components was at similar levels (1.20 mg/100 g) and statistically higher compared to the control product (0.26 mg/100 g) (Table 3).

The current research has shown that reformulating the meat product recipe has significantly reduced its cholesterol content, compared to the control product (Table 3). The lowest cholesterol content was found in group P_2_, with 60% plant components. The results of the study can be considered significant and beneficial to consumer health. According to current knowledge, the effect of dietary cholesterol intake on blood cholesterol levels in people with normal lipid metabolism is limited. However, in people with lipid disorders, limiting dietary cholesterol intake may have a positive effect on the lipid profile and overall health. Elevated blood cholesterol levels are one of the main risk factors for cardiovascular disease.

The total ash content in food products reflects its mineral content [64,71], which has been confirmed in the current study (Table 3 and Table 5).

The proper functioning of the human body depends on maintaining adequate levels of minerals in the diet. The most important of these are potassium, magnesium, calcium, phosphorus, iron, manganese, and selenium. They perform important functions in many physiological processes, including in the nervous, cardiovascular, skeletal, and immune systems. The results of the studies have shown that the use of plant components, rich in minerals, contributed to a significant enrichment of the mineral composition of the reformulated products. Products from research groups P_1_ and P_2_ were characterized by a statistically significantly higher content of potassium, magnesium, sodium, calcium, and micronutrients such as iron and manganese (*p* ≤ 0.05) compared to the control product (Table 5). The product from the P_2_ research group, with a higher proportion of plant components, including 40% chickpeas, had a higher content of zinc and other minerals compared to the product from the P_1_ group and the control group. The studies by Asmare and Admassu [65] also showed higher Na, K, Ca, Mg, and Fe content in sausage with added chickpea flour compared to the control group. The studies by Riadinskaja et al. [72] indicate that legumes (lentils, chickpeas, beans) are a good source of macro- and microelements, which was confirmed in poultry-based meat and vegetable preserves. Cocan et al. [73] confirmed the beneficial effect of adding pepper processing by-products on the mineral content of pork sausages.

The microbiological safety of meat products is determined primarily by the total number of mesophilic aerobic bacteria and the presence of *Pseudomonas* Spp. bacteria. These microorganisms are the main factors causing product spoilage, acting through protein proteolysis and the production of secondary metabolites that induce physicochemical changes in the product [72]. The effects of their activity include deterioration of sensory parameters, such as unpleasant odor or color change. The microbiological quality of plant products is usually determined based on the total number of microorganisms. Yeasts and molds play a significant role in the spoilage of plant products [63]. Our research has shown that poultry products containing plant components were microbiologically safe regardless of the research group. The total number of microorganisms in poultry products from group P_1_ ranged from 3.12 log cfu·g^−1^ to 3.16 log cfu·g^−1^ in products from group P_2_ (Table 3). The good microbiological quality of meat products containing plant components was also evidenced by the low level of *Pseudomonas* spp. bacteria (<1.0 ± 0.00). The significantly lower microbiological load in the test groups compared to the control product (3.38 log cfu·g^−1^) may have resulted from the content of fermented black garlic and dried tomatoes in the recipe. Skwarek and Karwowska [66] confirmed a reduction in the microbial load of fermented sausage containing tomato pomace. According to research by Augustyńska-Prejsnar et al. [70], the presence of black garlic in the recipe prevents the growth of microorganisms and increases the shelf life of poultry products when it during storage. This effect is related to the content of compounds with strong antimicrobial properties, mainly allicin [72,73]. Allicin, a reactive sulfur compound present in black fermented garlic and formed from alliin by the action of alliinase, exhibits strong antimicrobial properties. Its action is primarily based on a reaction with the thiol groups of proteins, which leads to damage to the cell membrane and inhibition of key metabolic enzymes. In addition, allicin can induce oxidative stress, damaging lipids, proteins, and DNA, as well as disrupting replication and transcription processes, effectively limiting the growth and survival of microorganisms. The bioactive effects of black garlic, including antimicrobial activity, are confirmed by the work of Mahros et al. [74] and Javed et al. [75].

An important issue in the production of health-promoting and enriched foods is the preservation of appropriate sensory characteristics so that enriched meat products do not differ from products made according to traditional recipes and are accepted by consumers. Sensory evaluation plays a key role in assessing the quality and acceptability of innovative products [52,69]. In terms of the desirability of smell, taste, and color, products containing plant additives, regardless of the test group, received significantly (*p* ≤ 0.05) higher ratings compared to the poultry meat product (Table 6).

Sensory scale: Odor: 5—highly desirable, strong, 4—desirable, strong; 3—moderately desirable, 2—slightly undesirable; 1—very undesirable, too intense/imperceptible; Flavor: 5—very desirable, 4—desirable 3—moderately desirable, 2—less desirable; 1—very undesirable; Desirability of color: 5—very desirable, uniform, 4—desirable, less uniform; 3—moderately desirable, uneven, 2—slightly undesirable, uneven; 1—very undesirable, uneven, altered in places; Connection: 5—very desirable, uniform, 4—desirable, uniform, 3—moderately desirable, uneven, 2—slightly undesirable, uneven, 1—very undesirable, uneven; Juiciness/moisture: 5—very desirable, moist, 4—desirable, slightly moist; 3—moderately desirable, too dry/too moist, 2—slightly undesirable, dry/moist; 1—very undesirable, very dry/wet; Consistency/structure: 5—very desirable, compact, smooth, homogeneous, 4—desirable, compact, homogeneous, 3—moderately desirable, less compact, heterogeneous, 2—slightly undesirable, too weak/too strong; 1—very undesirable, weak/strong; Spreadability: 5—very desirable, 4—desirable, 3—moderately desirable, 2—slightly undesirable; 1—very undesirable; General acceptability: 5—very desirable, 4—desirable, 3—moderately desirable, 2—slightly undesirable; 1—very undesirable

Within the test groups, spreadability, aroma, and color desirability were rated higher in group P_2_ with a higher proportion of chickpeas. In paste-type products, spreadability is important for even distribution of the product on bread. Raziuddin et al. [61] demonstrated that the addition of paprika to goat meat paste reduced the spreadability of the product but did not reduce its quality. In turn, Pathireje et al. [14] found that legumes in various forms are used to reformulate meat products as a binder, creating complex gel networks with meat proteins, which was confirmed in our own research by the improved spreadability of the P_2_ group product. In the studies by Asmare and Admassu [65], it was shown that dry fermented sausages with the addition of 20% chickpea flour had the most acceptable hedonic scale range. Similarly, in the studies by Ryadinskaya et al. [72], meat and vegetable preserves based on poultry meat with the addition of legumes received high marks in consumer evaluation. Our research showed that the taste of meat and vegetable products with cooked chickpeas in both the P_1_ and P_2_ groups was highly rated by the panelists. The reformulation methods proposed in our research did not confirm the concerns of Pathiraje et al. [14] that the flavor profile of legumes, which is undesirable to consumers, is associated with the presence of, for example, aldehydes, ketones, sulfur compounds, and saponins. In the conducted studies, the conciseness (connection) of the poultry meat product with plant components was rated higher (4.65–4.70 points) compared to the control group product. These results indicate that both the degree of grinding of plant components, where chickpeas were the main component, and the optimal proportions of meat and plant components had a positive effect in obtaining a uniform and consistent paste structure. The grinding of steamed chickpeas and other plant ingredients into fine particles increased the surface area of the particles and may have improved the cohesion of the mixture. The protein and starch contained in chickpeas gel during heat treatment, creates a structure that binds meat and plant ingredients together. In addition, steaming in water and slow cooling promote the stabilization of the protein-starch network, which may have contributed to the effect obtained, despite the higher fiber content often associated with products containing plant components [22,23].

## 4. Conclusions

The results of the study indicate that combining poultry meat and plant components (chickpeas, red peppers, sun-dried tomatoes, fermented black garlic, linseed oil, hemp oil) is a technologically feasible alternative that allows for the creation of products with increased nutritional value, without the use of synthetic additives.

The results of the study confirm improvements in the health-promoting properties of poultry products in the form of a paste with plant components, regardless of the test group, including a reduction in fat content while maintaining a favorable fatty acid profile, an increase in fiber content, a reduction in cholesterol content, and an increase in mineral content (Mg, Mn, K, Na, Ca, Fe) compared to the control product (classic chicken pâté in paste form) while maintaining good quality, microbiological safety, and sensory acceptability.

Products containing plant components had a higher pH and darker color (higher degree of red and yellow color saturation) compared to the control product.

Within the test groups, the product from group P_2_, with a higher proportion of plant components, was rated higher in terms of taste, aroma, and spreadability. The product from group P_2_, containing 60% plant components, had a more favorable omega-6 to omega-3 ratio, more fiber, Mg, Na, Ca, Zn, and a darker color compared to the product from group P_1_, containing 40% plant components.

The results indicate the high industrial potential of such poultry products as an alternative to traditional meat products, offering nutritional benefits, including a healthy lipid profile, fiber and minerals enrichment, and the possibility of introducing natural health-promoting components. Pilot studies and production process scalability are recommended as research directions, including the integration of the formula with industrial lines, the assessment of the repeatability of technological parameters, and production costs. The implementation of this type of product into industrial practice may contribute to the development of the hybrid food segment, combining traditional poultry meat with valuable plant components, while responding to the needs of consumers looking for healthier and more sustainable food alternatives.

## 5. Patents

A patent application has been filed with WIPO ST 10/CPL446536.

## Figures and Tables

**Table 1:** Recipe composition of a poultry product containing plant components (%).

Ingredients		Test Groups		
		Group P_M_	Group P_1_	Group P_2_
Meat-based raw material:				
Breast meat of slaughter turkey		60.00	50.00	30.00
Pork jowl		15.00	0.00	0.00
Plant-based components	Wheat roll	10.00	0.00	0.00
	Onion	3.00	3.00	3.00
	Garlic	2.00	2.00	2.00
	Chickpeas	0.00	20.00	40.00
	Red pepper	0.00	7.00	7.00
	Flaxseed oil	0.00	4.00	4.00
	Hemp oil	0.00	3.00	3.00
	Dried tomatoes	0.00	0.50	0.50
	Fermented black garlic	0.00	0.50	0.50
Black pepper		0.20	0.20	0.20
Non-iodised salt		0.80	0.80	0.80
Ice water		9.00	9.00	9.00

Group P_M_—poultry pâté in paste form (75% meat content); P_1_—poultry product in paste form with 50% slaughter turkey meat and 40% plant components; P_2_—poultry product in paste form with 30% slaughter turkey meat and 60% plant components.

**Table 2:** Physical characteristics of poultry products with varying proportions of plant components ($\bar{x}$ ± SD).

Parameter	Poultry Products			
	Group P_M_	Group P_1_	Group P_2_	*p* Value
pH	5.86 ^a^ ± 0.01	6.12 ^b^ ± 0.02	6.18 ^b^ ± 0.01	0.0023
Color cross-section:				
L*—lightness	79.03 ^a^ ± 1.89	66.82 ^b^ ± 3.24	62.46 ^c^ ± 4.67	0.000
a*—redness	6.26 ^b^ ± 0.23	13.60 ^a^ ± 0.68	14.07 ^a^ ± 0.77	0.001
b*—yellowness	16.27 ^c^ ± 0.48	29.39 ^b^ ± 1.93	32.79 ^a^ ± 1.54	0.000

Group P_M_—poultry pâté in paste form (75% meat content); P_1_—poultry product in paste form with 50% slaughter turkey meat and 40% plant components; P_2_—poultry product in paste form with 30% slaughter turkey meat and 60% plant components; values marked with the same letter do not differ significantly at *p* ≤ 0.05.

**Table 3:** Nutritional value and microbiological load of poultry products with varying proportions of plant components ($\bar{x}$ ± SD).

Parameter	Poultry Products			*p* Value
	Group P_M_	Group P_1_	Group P_2_	
Protein (N × 6.25)	17.70 ± 2.20	16.72 ± 1.60	15.07 ± 2.40	0.051
Fat (%)	13.06 ^a^ ± 2.06	8.86 ^b^ ± 1.56	8.16 ^b^ ± 1.36	0.033
Total ash (%)	1.17 ^c^ ± 0.45	1.22 ^b^ ± 0.20	1.48 ^a^ ± 0.32	0.014
Fiber (%)	1.01 ^c^ ± 0.15	3.08 ^b^ ± 0.13	4.44 ^a^ ± 0.10	0.002
Tocopherol content dl alfa-tokoferol mg/100 g	0.26 ^b^ ± 0.06	1.20 ^a^ ± 0.05	1.21 ^a^ ± 0.50	0.004
Cholesterol mg/100 g	58.70 ^a^ ± 3.12	27.98 ^b^ ± 1.86	20.56 ^c^ ± 2.15	0.000
Total number of bacteria (log cfu·g^−1^)	3.34 ^a^ ± 0.04	3.12 ^b^ ± 0.02	3.16 ^b^ ± 0.01	0.003
*Pseudomonas* Spp.	<0.10	<0.10	<0.10	*-*

Group P_M_—poultry pâté in paste form (75% meat content); P_1_—poultry product in paste form with 50% slaughter turkey meat and 40% plant components; P_2_—poultry product in paste form with 30% slaughter turkey meat and 60% plant components; values marked with the same letter do not differ significantly at *p* ≤ 0.05.

**Table 4:** Fatty acid profile of poultry products with varying proportions of plant components ($\bar{x}$ ± SD).

Parameter (%)	Poultry products			*p* Value
	Grupa P_M_	Grupa P_1_	Grupa P_2_	
SFA				
Lauric acid C 12:0	0.08 ± 0.02	<0.05	<0.05	
Tetradecanoic acid C 14:0	0.83 ^a^ ± 0.03	0.08 ^b^ ± 0.01	0.07 ^b^ ± 0.01	0.000
Pentadecanoic acid C 15:0	0.05 ± 0.01	<0.05	<0.05	
Hexadecanoic acid C 16:0	19.41 ^a^ ± 0.25	7.18 ^b^ ± 0.35	6.76 ^c^ ± 0.63	0.000
Heptadecanoic acid C 17:0	0.19 ^a^ ± 0.02	0.08 ^b^ ± 0.01	0.07 ^b^ ± 0.01	0.001
Octadecanoic acid C 18:0	8.82 ^a^ ± 0.14	4.42 ^b^ ± 0.26	4.00 ^b^ ± 0.28	0.002
Eicosanoic acid C 20:0	0.35 ^c^ ± 0.02	0.51 ^a^ ± 0.03	0.44 ^b^ ± 0.02	0.021
Heneicosanoic acid C 21:0	<0.05	<0.05	<0.05	-
Tricosanoic acid C 23:0	<0.05	<0.05	<0.05 ±	-
Tetracosanoic acid C 24:0	0.12 ± 0.01	0.15 ± 0.01	0.14 ± 0.01	0.165
Σ saturated acids	2.49 ^a^ ± 0.03	1.27 ^b^ ± 0.10	1.29 ^b^ ± 0.22	0.021
MUFA				
Oleomyristic acid C 14:1	<0.05	<0.05	<0.05	-
Hexadecanoic acid C 16:1	1.97 ^a^ ± 0.02	0.25 ^b^ ± 0.01	0.23 ^b^ ± 0.01	0.022
Heptadecenoic acid C 17:1	0.16 ^a^ ± 0.01	0.05 ^b^ ± 0.05	0.06 ^b^ ± 0.01	0.005
Cis-9-octadecenoic acid C 18:1	46.29 ^a^ ± 0.56	27.74 ^c^ ± 1.41	29.15 ^b^ ± 0.94	0.000
Eicozenic acid C 20:1	0.97 ^a^ ± 0.01	0.31 ^b^ ± 0.01	0.32 ^b^ ± 0.02	0.001
Tetracosoic acid C 24:1	0.09 ^a^ ± 0.01	0.11 ^b^ ± 0.00	0.09 ^b^ ± 0.00	0.011
Σ monounsaturated fatty acids	4.35 ^a^ ± 0.14	2.99 ^c^ ± 0.21	3.40 ^b^ ± 0.75	0.000
PUFA				
Eicosadienoic acid C 20:2	0.39 ^a^ ± 0.01	0.06 ^b^ ± 0.01	0.05 ^b^ ± 0.00	0.002
Eicosatrienic acid C20:3 n-3	0.06 ± 0.01	<0.05	<0.05	
Σ polyunsaturated fatty acids	1.42 ^c^ ± 0.10	5.90 ^b^ ± 0.12	6.43 ^a^ ± 1.88	0.000
Fatty acids N-3	0.20 ^c^ ± 0.01	2.39 ^b^ ± 0.20	3.19 ^a^ ± 1.09	0.000
Fatty acids N-6	1.66 ^b^ ± 0.03	3.25 ^a^ ± 0.01	3.20 ^a^ ± 0.78	0.000
TRANS				
Trans-hexadecenoic acid C 16: 1 trans				
Σ trans fatty acids	<0.10	<0.10	<0.10	-

SFA (Saturated Fatty Acids), MUFA (Monounsaturated Fatty Acids), PUFA (Polyunsaturated Fatty Acids); Group P_M_—poultry pâté in paste form (75% meat content); P_1_—poultry product in paste form with 50% slaughter turkey meat and 40% plant components; P_2_—poultry product in paste form with 30% slaughter turkey meat and 60% plant components; values marked with the same letter do not differ significantly at *p* ≤ 0.05.

**Table 5:** Mineral content in poultry products with varying proportions of plant components ($\bar{x}$ ± SD).

Parameter (mg/kg)	Poultry Products			*p* Value
	Group M	Group P_1_	Group P_2_	
Phosphorous	1658.00 ^a^ ± 45.52	1283.80 ^b^ ± 42.72	1133.80 ^c^ ± 64.63	0.002
Magnesium	204.00 ^c^ ± 21.12	259.20 ^b^ ± 7.12	271.40 ^a^ ± 13.15	0.006
Manganese	0.31 ^c^ ± 0.15	3.75 ^b^ ± 0.09	5.08 ^a^ ± 0.25	0.000
Potassium	1949.00 ^c^ ± 62.10	2077.60 ^a^ ± 67.92	1989.40 ^b^ ± 92.57	0.011
Sodium	2378.20 ^c^ ± 75.59	2428.00 ^b^ ± 78.92	3046.20 ^a^ ± 96.46	0.003
Calcium	102.00 ^c^ ± 12.24	315.00 ^b^ ± 5.48	364.80 ^a^ ± 15.34	0.000
Iron	6.92 ^b^ ± 1.01	9.56 ^a^ ± 1.56	9.24 ^a^ ± 1.04	0.000
Zinc	11.12 ^b^ ± 0.11	11.60 ^b^ ± 0.46	12.88 ^a^ ± 1.81	0.033

Group P_M_—poultry pâté in paste form (75% meat content); P_1_—poultry product in paste form with 50% slaughter turkey meat and 40% plant components; P_2_—poultry product in paste form with 30% slaughter turkey meat and 60% plant components; values marked with the same letter do not differ significantly at *p* ≤ 0.05.

**Table 6:** Sensory characteristics of poultry products with varying proportions of plant components (points) ($\bar{x}$ ± SD).

Parameter	Poultry Products			*p* Value
	Group P_M_	Group P_1_	Group P_2_	
Odor	3.60 ^b^ ± 0.52	4.65 ^a^ ± 0.41	4.75 ^a^ ± 0.42	0.032
Flavor	3.80 ^c^ ± 0.48	4.40 ^b^ ± 0.39	4.85 ^a^ ± 0.34	0.021
Desirability of color	3.00 ^c^ ± 0.33	4.60 ^b^ ± 0.39	4.75 ^a^ ± 0.42	0.041
Connection	4.00 ^b^± 0.41	4.65 ^a^ ± 0.34	4.70 ^a^ ± 0.42	0.036
Juiciness/moisture	3.00 ^b^ ± 0.47	4.50 ^a^ ± 0.47	4.65 ^a^ ± 0.63	0.002
Consistency/structure	4.40 ± 0.39	4.35 ± 0.41	4.15 ± 0.24	0.052
Spreadability	3.00 ^c^ ± 0.53	4.40 ^b^ ± 0.46	4.85 ^a^ ± 0.34	0.004
General acceptability	3.85 ^c^ ± 0.67	4.50 ^b^ ± 0.47	4.70 ^a^ ± 0.42	0.011

Group P_M_—poultry pâté in paste form (75% meat content); P_1_—poultry product in paste form with 50% slaughter turkey meat and 40% plant components; P_2_—poultry product in paste form with 30% slaughter turkey meat and 60% plant components; values marked with the same letter do not differ significantly at *p* ≤ 0.05.

## Data Availability

The datasets presented in this article are not readily available because the data are part of an ongoing study. Requests to access the datasets should be directed to the corresponding author.
